# Supplementary figures and images for: New in vitro system to predict chemotherapeutic efficacy of drug combinations in fresh tumor samples
Source: PeerJ. 2017 Mar 2;5:e3030. doi: 10.7717/peerj.3030 (PMC5337084; doi:10.7717/peerj.3030)

**A**

### Carbo & Pac

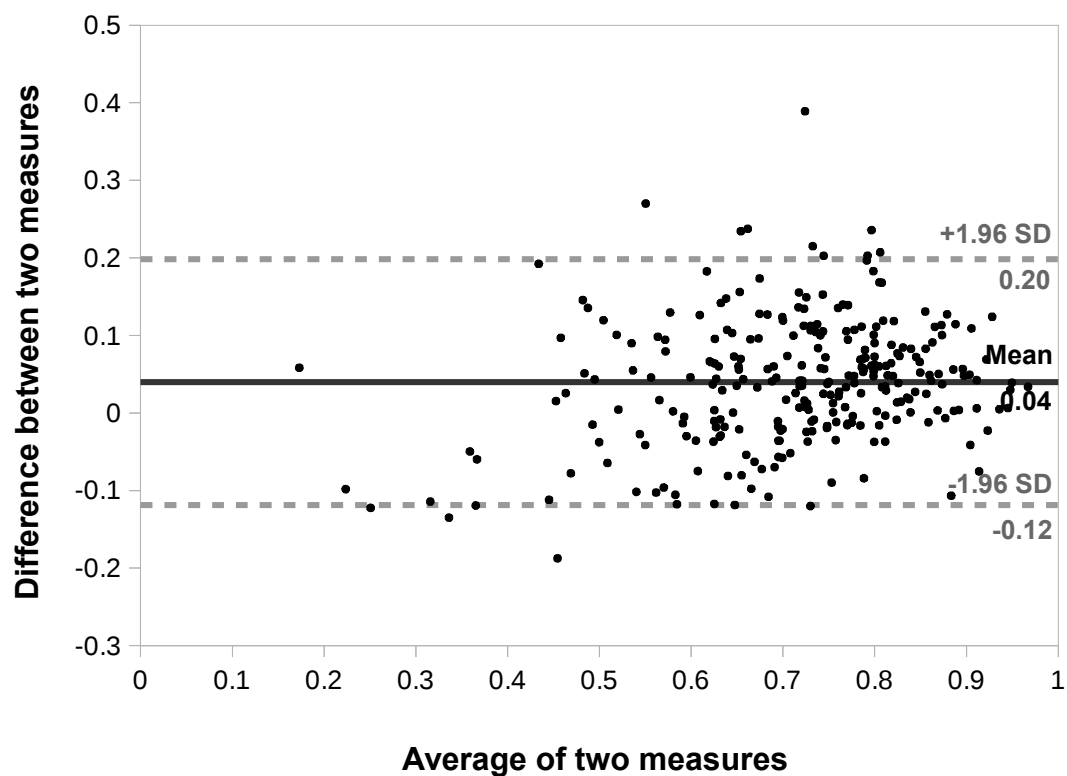

**B**

### Carbo & Caelyx

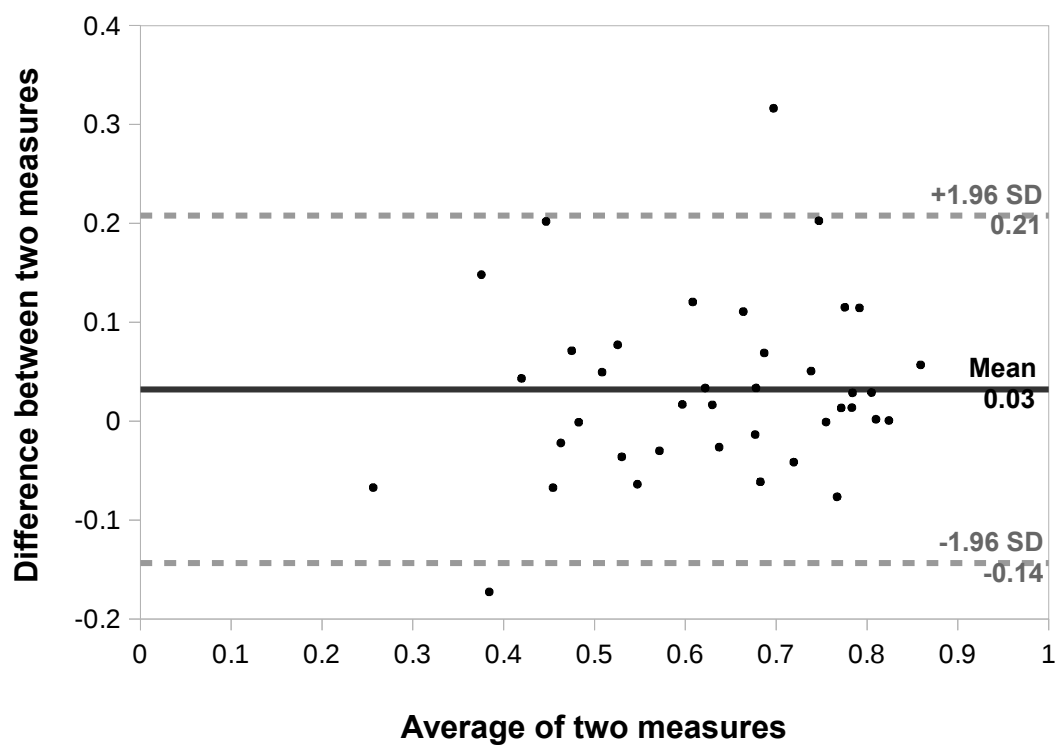

**C**

### Carbo & Doxo

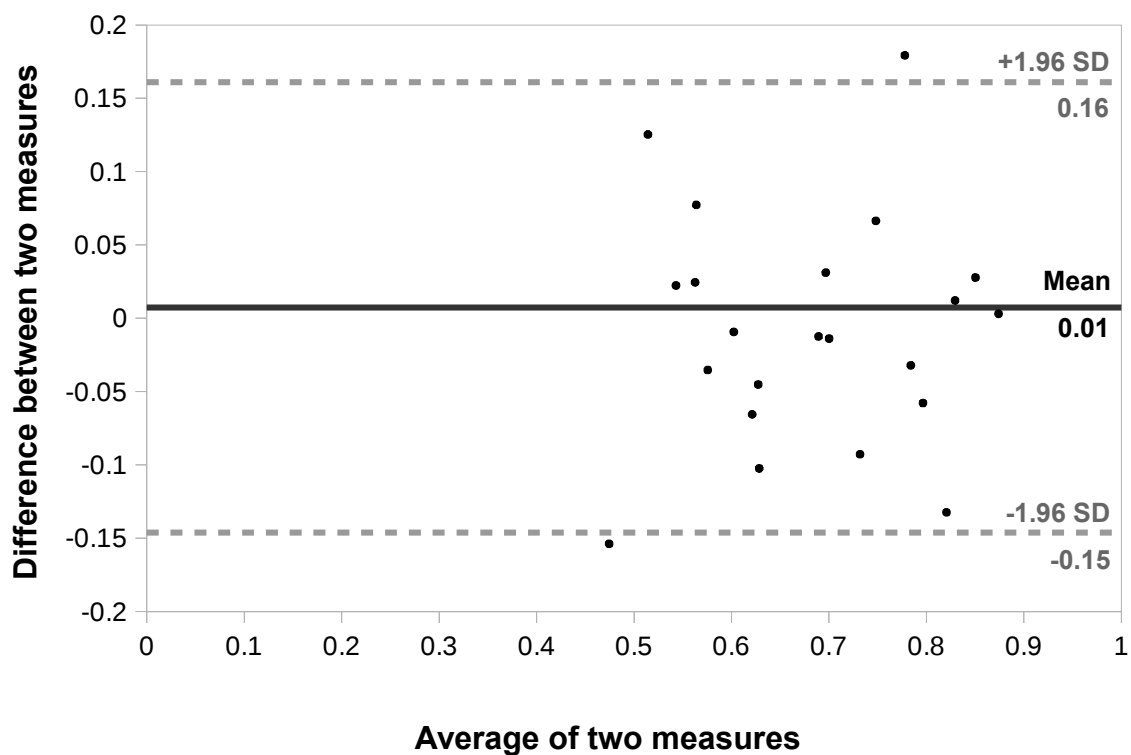

**D**

### Carbo & Etop

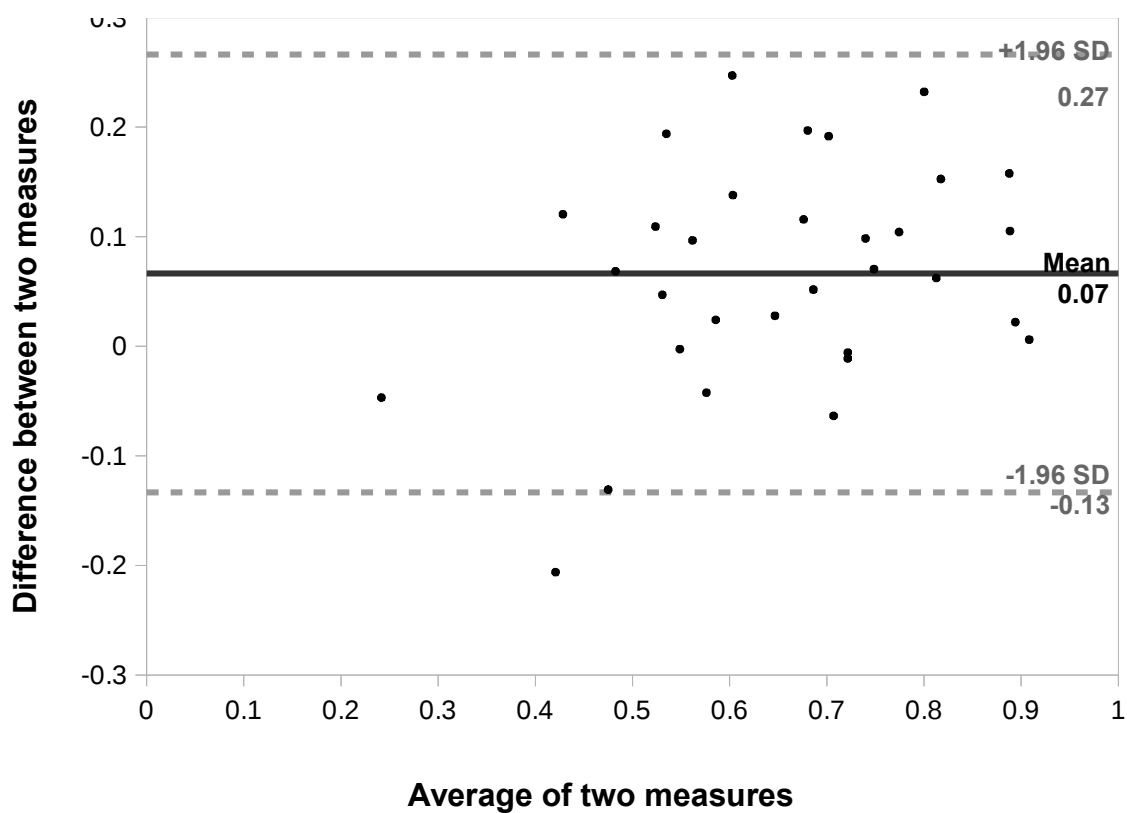

**E**

# **Carbo & Topo**

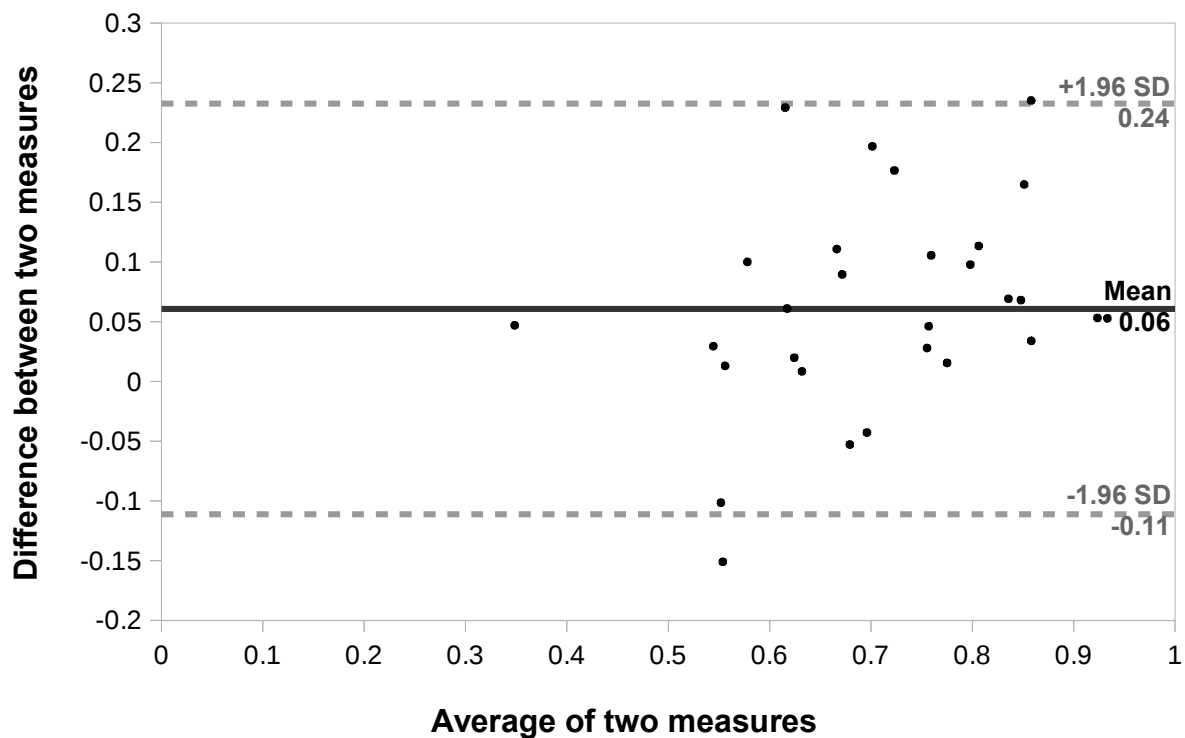

**F**

# **Carbo & Doce**

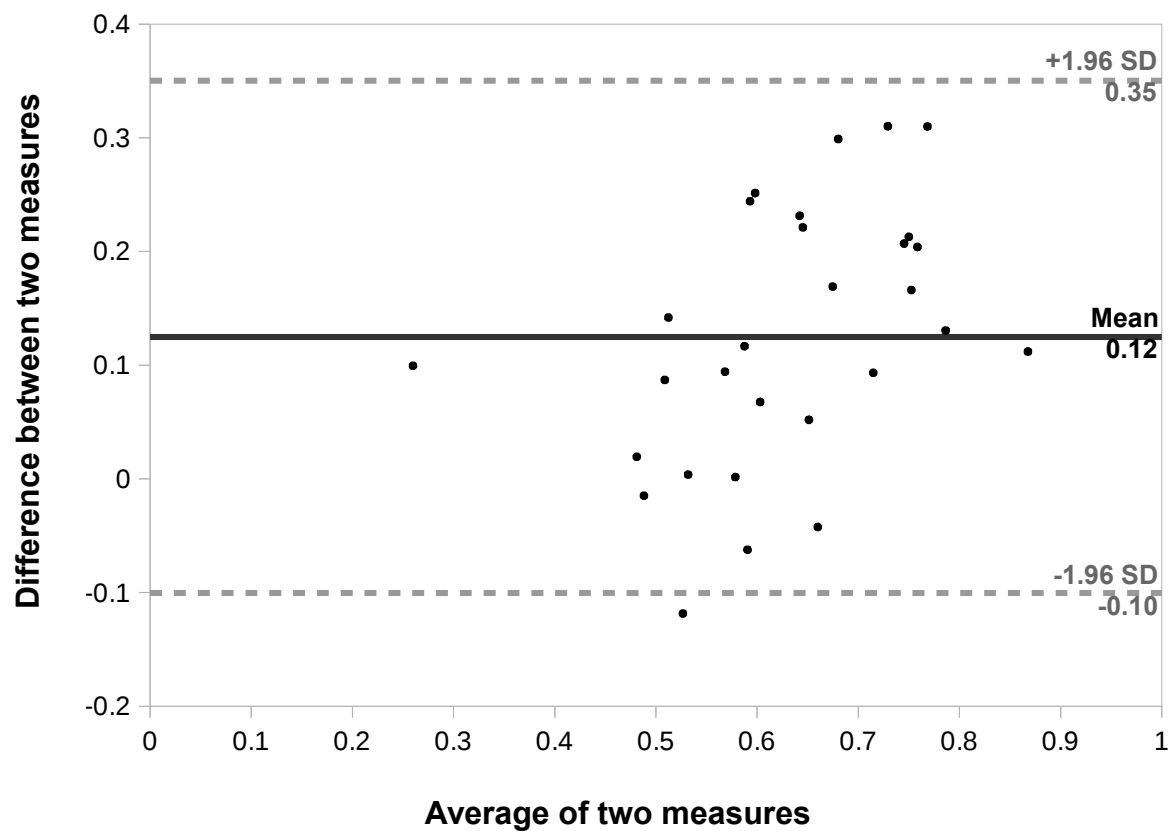

**G**

# **Carbo & Gem**

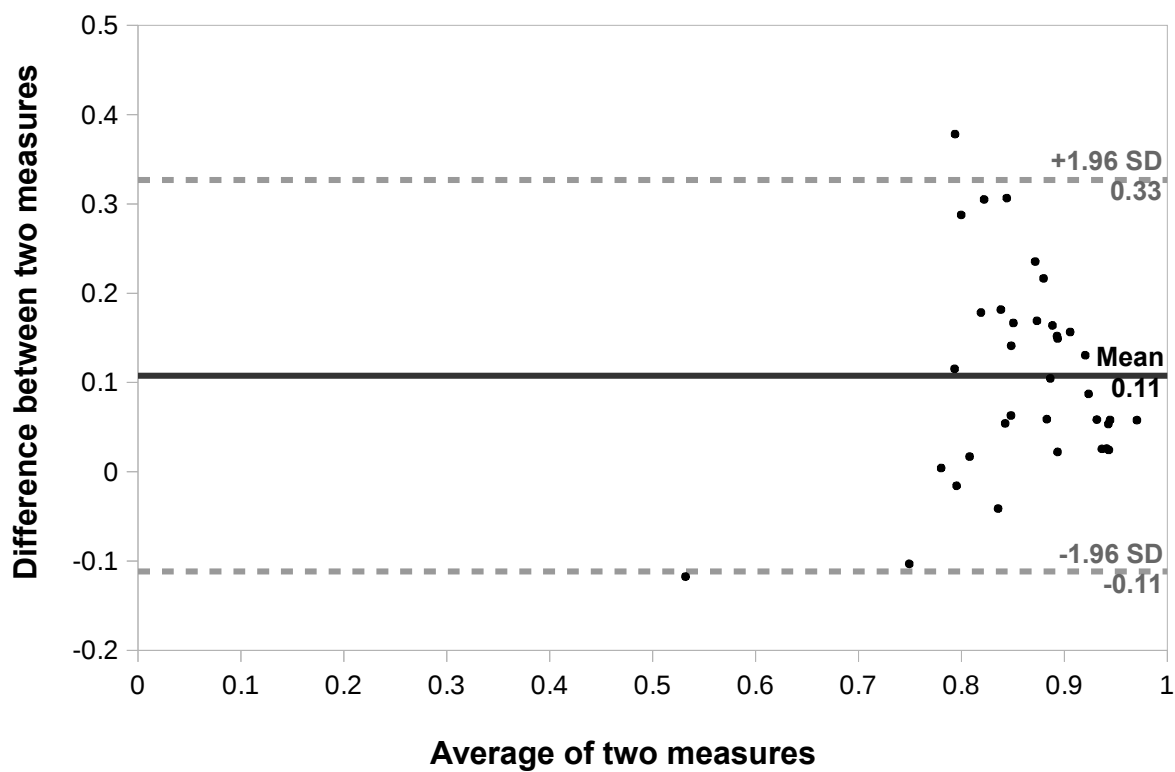

**H**

# **5FU & SN38**

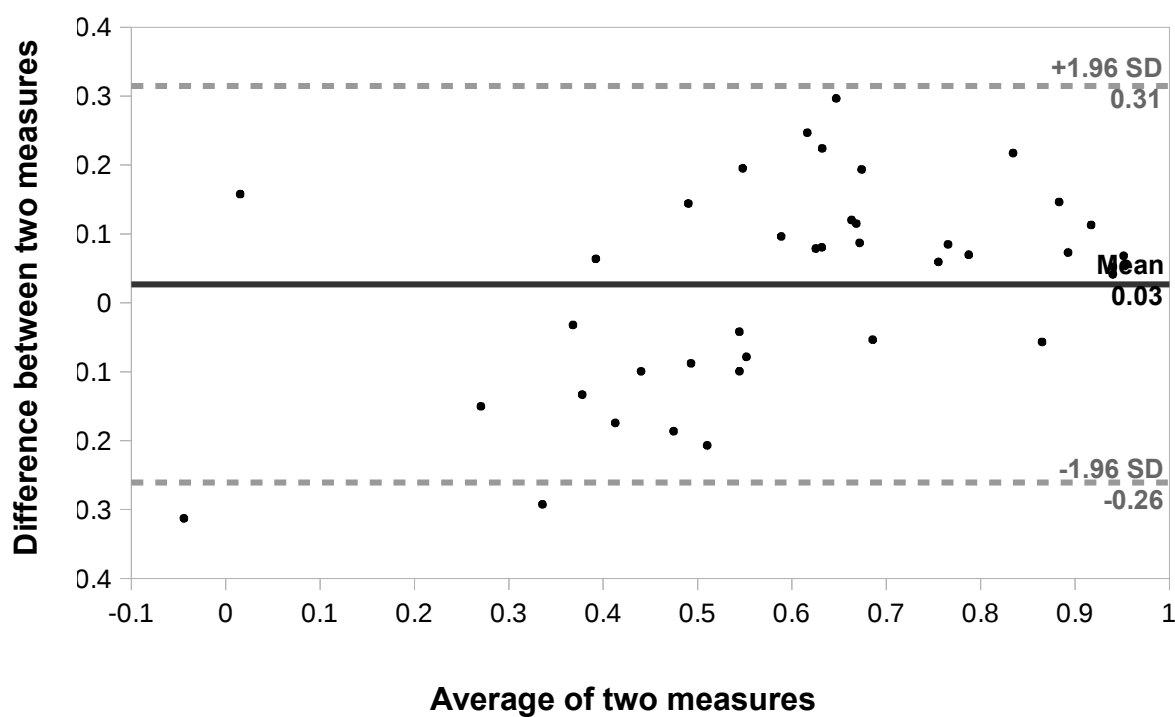

I

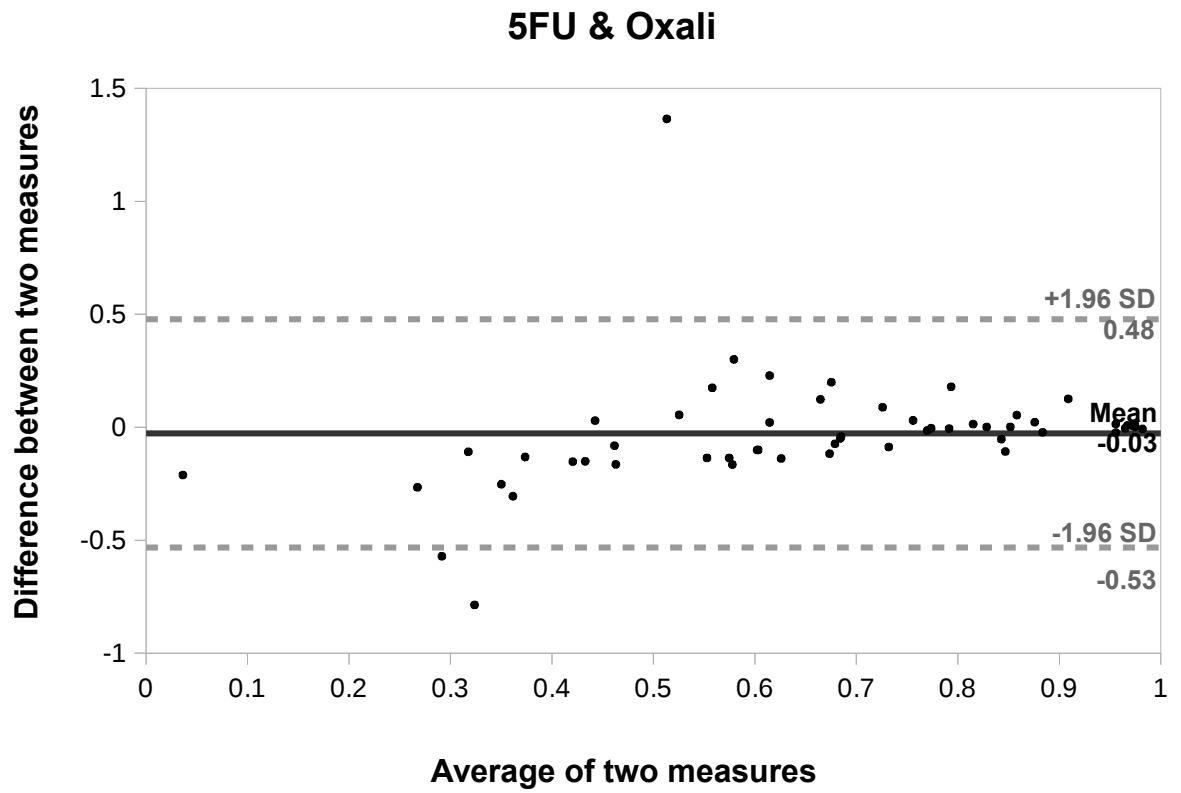

Supplement: Figure S1 — (A–I) Measured and calculated PCI values of the different carboplatin (A–G) and 5-fluorouracil (H and I) combinations were shown in a Bland-Altman-Plot to provide an adequate description of agreement. [file peerj-05-3030-s001.pdf]
