# Supplementary material for: New in vitro system to predict chemotherapeutic efficacy of drug combinations in fresh tumor samples
Source: PeerJ. 2017 Mar 2;5:e3030. doi: 10.7717/peerj.3030 (PMC5337084; doi:10.7717/peerj.3030)
Supplement: Supplementary file 2 [file peerj-05-3030-s002.odt]

|  | Carbo & Pac | Carbo & Caelyx | Carbo & Doxo | Carbo & Etop | Carbo & Topo | Carbo & Doce | Carbo & Gem | 5FU & SN-38 | 5FU & Oxali |
| --- | --- | --- | --- | --- | --- | --- | --- | --- | --- |
| Carbo / 5FU (SD [%]) | 17.3 | 14.8 | 17.6 | 17.1 | 16.5 | 17.2 | 15.4 | 27.6 | 28.1 |
| Second Drug (SD [%]) | 18.5 | 21.0 | 18.5 | 21.4 | 17.9 | 19.9 | 12.9 | 26 | 17 |
| Measured (SD [%]) | 14.9 | 16.3 | 15.3 | 18.3 | 15.4 | 15.7 | 10.3 | 27.9 | 23.7 |
| Calculated (SD [%]) | 13.1 | 14.8 | 13.4 | 14.9 | 12.8 | 12.1 | 8.9 | 21.1 | 19.8 |
